# EXPLORING OLDER ADULTS’ EXPERIENCES IN CARE TRANSITIONS

**DOI:** 10.1093/geroni/igz038.3500

**Published:** 2019-11-08

**Authors:** Flora Vieira Zamora, Michelle Silver, Whitney Berta, Michelle Nelson, Angela Hamilton

**Affiliations:** University of Toronto, Toronto, Ontario, Canada

## Abstract

Older adults are Canada’s largest growing demographic. Later adulthood is frequently accompanied by increased comorbidities, resulting in more people living with chronic conditions for longer portions of their lives and requiring care across multiple settings. These individuals are also the most susceptible to challenges within health care systems, especially during vulnerable times such as care transitions, which can be challenging due to issues of care integration and coordination. A scoping review was conducted to explore the experiences of older adults transitioning through various levels of care. The main themes found included personal realizations, social connectedness, importance of navigating the system and recommendations for the future. During care transitions, older adults must carefully consider their personal circumstances and limitations and often accept a new baseline, thus, adapting their lives and activities to match possible limitations. Older adults indicate the need for strong social networks, accessible and available services, as well as effective communication, information, education and engagement during care transitions. Issues with care transitions can be exacerbated in smaller communities, where resources and services may be limited. As such, this scoping review is the foundation for an ongoing systematic review which aims to summarize what is known about care transitions for older adults living in small and rural communities. By better understanding the different interacting factors that might influence care transitions for older adults living in small communities, important and sustainable changes can be identified and implemented to ensure that care transitions for older adults are safe, positive and empowering.

